# Lateral Extra‐articular Tenodesis Using Knotless All‐Suture Anchor Fixation With Suture Tape: A Modified Technique for Augmenting Anterior Cruciate Ligament Reconstruction

**DOI:** 10.1002/atn2.70023

**Published:** 2026-05-04

**Authors:** Bryson R. Kemler, Seena Sebt, Michael B. Banffy

**Affiliations:** ^1^ Cedars Sinai Medical Center Los Angeles California U.S.A.

## Abstract

Lateral extra‐articular tenodesis has emerged as a valuable adjunct to anterior cruciate ligament reconstruction for mitigating residual rotational instability. We describe a modified lateral extra‐articular tenodesis technique utilizing an all‐suture knotless anchor with suture tape, which provides strong, flexible fixation with reduced soft‐tissue trauma. This technique improves graft integration, minimizes hardware prominence, and offers superior biomechanical stability compared with traditional fixation methods.

## Graphical abstract

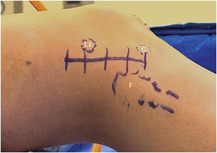

**VIDEO 1:** Right knee, lateral side. Right lateral femur, proximal and posterior to the lateral epicondyle. This video describes a modified lateral extra‐articular tenodesis technique for augmenting anterior cruciate ligament reconstruction utilizing knotless‐all suture anchor fixation with suture tape. Video content can be viewed at https://doi.org/10.1002/atn2.70023.

Anterior cruciate ligament (ACL) reconstruction is a widely performed surgical procedure aimed at restoring knee stability following ACL injury. While standard ACL reconstruction effectively addresses anterior tibial translation, residual rotational instability remains a challenge in certain patient populations—particularly those with high‐grade pivot shift, generalized ligamentous laxity, revision ACL surgery, or high‐risk sports participation.^1^, ^2^

To address these concerns, lateral extra‐articular tenodesis (LET) has re‐emerged as a viable augmentation technique. Originally described by Lemaire in the 1970s, LET functions by reinforcing the anterolateral structures of the knee, thereby reducing excessive internal tibial rotation.^2^, ^3^ Multiple studies have showed that combining LET with ACL reconstruction significantly reduces graft failure rates, particularly in high‐risk populations.^1^, ^2^, ^4^, ^5^, ^6^, ^7^, ^8^ The recent revival of LET techniques has been supported by evidence showing that restoring lateral knee stability enhances overall biomechanical outcomes.^6^, ^9^, ^10^, ^11^

Traditional LET procedures often involve the use of rigid fixation methods such as interference screws, bone tunnels, or cortical buttons. However, these approaches can lead to overconstraint of the knee, increased hardware prominence, and potential soft‐tissue irritation.^3^, ^12^ To overcome these limitations, all‐suture knotless anchor fixation has emerged as a promising alternative.^13^, ^14^, ^15^, ^16^ Knotless suture anchors offer several advantages over conventional fixation methods, making them an attractive option for LET. Their minimally invasive nature reduces iatrogenic trauma to soft tissues, promoting a less disruptive surgical approach.^14^, ^15^ Additionally, they facilitate dynamic load distribution, allowing controlled micromotion that enhances graft integration and overall stability.^13^, ^14^ The low‐profile design of these anchors minimizes hardware prominence, reducing the risk of irritation and discomfort for the patient.^14^, ^16^ Furthermore, they provide biomechanical strength comparable to traditional fixation techniques without introducing excessive rigidity, thereby preserving natural knee movement.^3^, ^9^ Recent biomechanical studies suggest that LET with a knotless all‐suture anchor delivers superior rotational control while maintaining physiological knee motion, making it a promising alternative to conventional fixation strategies.^9^, ^14^, ^16^ Finally, incorporating suture tape into knotless all‐suture anchors may enhance repair strength by distributing loads more evenly across repaired tissue, as has been shown previously in biomechanical evaluation of the shoulder.^17^ This technique enables a balanced, dynamic stabilization of the lateral knee structures, preventing excessive tibial internal rotation without restricting natural joint movement.^10^, ^11^

This article and video describe a modified Lemaire LET technique utilizing knotless all‐suture anchor fixation, offering a minimally invasive, biomechanically optimized alternative to traditional LET fixation methods. The goal is to provide enhanced rotational stability, reduced complications, and improved long‐term outcomes in ACL reconstruction patients (Video 1).

## SURGICAL TECHNIQUE

### Patient Positioning and Graft Harvest

The patient is positioned supine on the operating table with standard sterile preparation and draping of the operative leg, ensuring adequate exposure of the lateral aspect of the knee. A well‐padded post is placed against the proximal thigh to facilitate valgus stress of the knee during the arthroscopic portion of the procedure. A tourniquet is applied but typically not inflated unless hemostasis is required. Arthroscopic ACL reconstruction is performed prior to the LET procedure.

**FIGURE 1:**
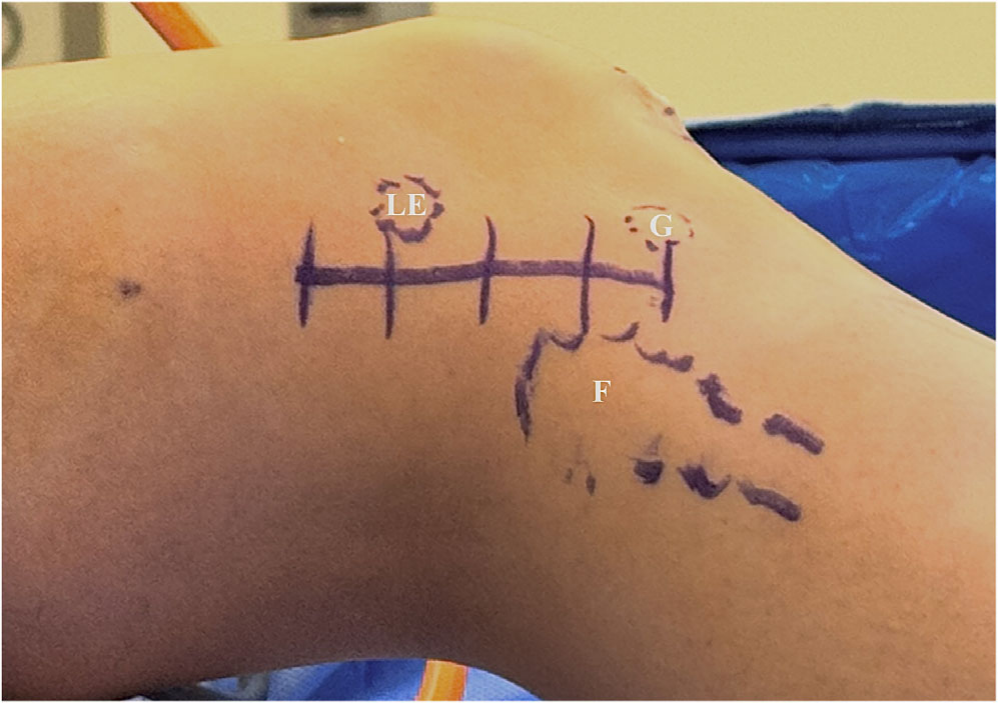
Right knee, lateral side. Right lateral femur, proximal and posterior to the lateral epicondyle. Once anterior cruciate ligament reconstruction is complete, the patient remains positioned supine with the knee flexed approximately 30° in preparation for lateral extra‐articular tenodesis. A 5 cm incision is made along the lateral aspect of the knee, using the lateral epicondyle (LE), Gerdy's tubercle (G), and the fibular head (F) as anatomic landmarks.

The incision begins approximately 1 cm proximal to the lateral epicondyle and is extended 5 cm to the midpoint between Gerdy's tubercle and the fibular head (Figure 1). Dissection is carried down to the level of the ilitiobial band (ITB). Using the lateral epicondyle as a landmark, a 1 cm strip of tendon corresponding roughly to the middle of the ITB is marked and harvested. Distally this is left attached to Gerdy's tubercle while proximally it is amputated 2 to 3 cm proximal to the lateral epicondyle.

### Graft Passage

**FIGURE 2:**
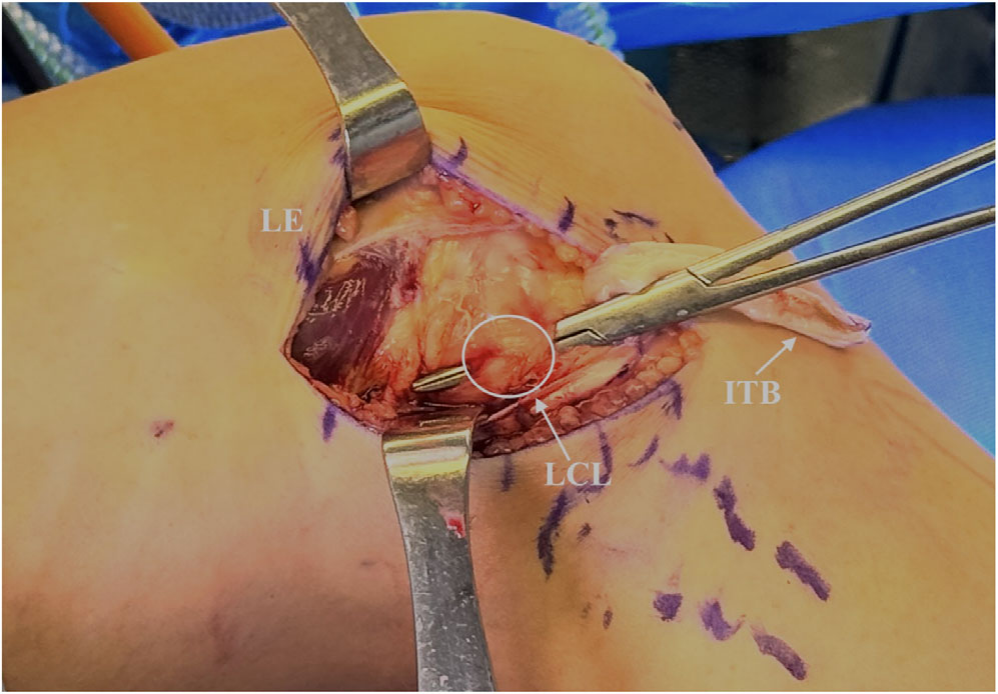
Right knee, lateral side. Right lateral femur, proximal and posterior to the lateral epicondyle. Following dissection through subcutaneous tissue, the ilitiobial band (ITB) is identified and a 1 cm strip of tendon corresponding roughly to the middle of the ITB is marked and harvested. Distally, this is left attached to Gerdy's tubercle while proximally it is amputated 2‐3 cm proximal to the lateral epicondyle. The lateral collateral ligament (LCL) is identified and an extra‐articular tissue plane is developed between the LCL and joint capsule. A Kelly clamp depicts the extra‐articular passage of the ITB graft deep to the ligament. (ITB, ilitiobial band; LCL, lateral collateral ligament; LE, lateral epicondyle.)

**FIGURE 3:**
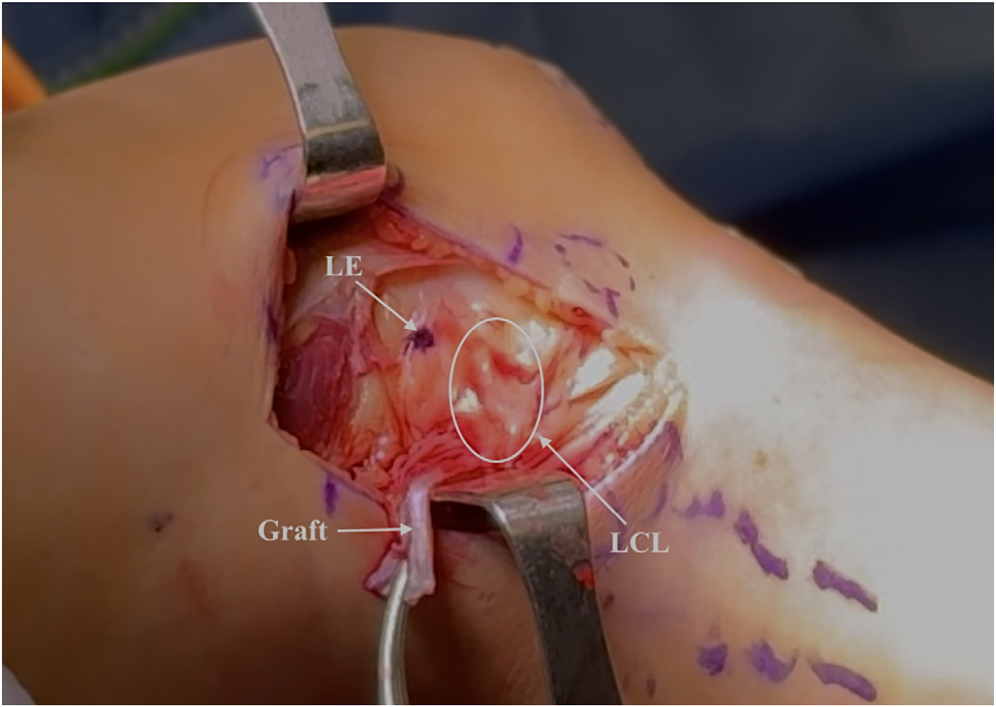
Right knee, lateral side. Right lateral femur, proximal and posterior to the lateral epicondyle. The graft is then passed distal to proximal in an extra‐articular fashion deep to the lateral collateral ligament (LCL). Following graft passage, the lateral epicondyle (LE) is palpated and marked. The femoral isometric point and site of anchor placement is 8 to 10 mm proximal and posterior to the LE. (LCL, lateral collateral ligament; LE, lateral epicondyle.)

An extra‐articular tissue plane between the lateral collateral ligament (LCL) and lateral capsule is then developed with a Metzenbaum scissor. A shuttle suture or Kelly clamp may be used to pass the graft distal to proximal (Figure 2). Precise identification of the anatomical insertion points is critical for achieving optimal biomechanical function. The lateral femoral epicondyle is therefore palpated, and the insertion site of the LCL is identified (Figure 3). The femoral isometric point for graft attachment is located approximately 8 to 10 mm proximal and posterior to the LCL attachment. This positioning allows the graft to mimic the function of the anterolateral ligament, providing rotational control without restricting physiological knee motion, and is the site of knotless all‐suture anchor fixation.

Proper tensioning of the graft is crucial to avoid overconstraint of the knee. The surgeon applies controlled tension while assessing tibial internal rotation and knee flexion to ensure a balanced graft placement.

### Fixation and Tensioning With Knotless All‐Suture Anchor

**FIGURE 4:**
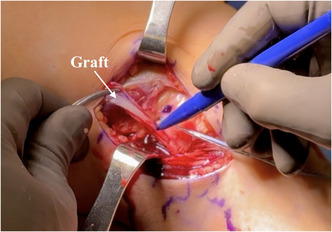
Right knee, lateral side. Right lateral femur, proximal and posterior to the lateral epicondyle. The 1.8 mm all‐suture anchor loaded with 1.4 mm suture tape and shuttle suture is inserted at the femoral isometric point 8 to 10 mm posterior and proximal to the LE. With the knee at 30° of flexion and neutral rotation, the graft is marked at the site of fixation prior to Krakow stitching. (LE, lateral epicondyle.)

**FIGURE 5:**
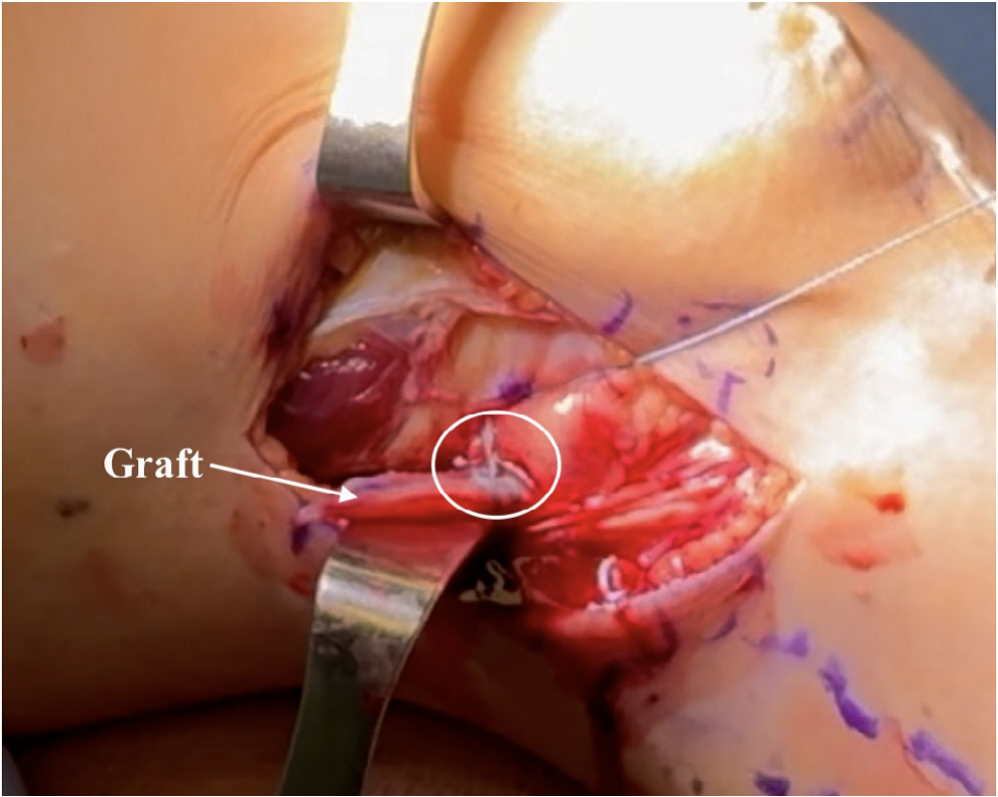
Right knee, lateral side. Right lateral femur, proximal and posterior to the lateral epicondyle. The graft is stitched in a Krakow orientation using the 1.4 mm suture tape, which is then shuttled through the anchor via the looped end of the shuttle suture. The knee remains set at 30° of flexion and neutral rotation while the graft is tensioned and fixed in an onlay fashion (circled) to the femur.

**FIGURE 6:**
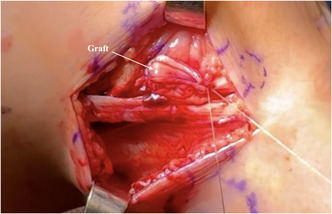
Right knee, lateral side. Right lateral femur, proximal and posterior to the lateral epicondyle. The excess graft is then doubled back and sutured on itself using #2 braided suture to reinforce the construct.

A drill guide is positioned at the femoral attachment site and is oriented approximately 25° anteriorly and 25° distally to avoid convergence with the previously drilled ACL tunnel.^18^ Once the pilot hole is created to accommodate the 1.8‐mm anchor (Q‐FIX Knotless All‐Suture Anchor, Smith & Nephew), the drill is cycled several times to clear bony debris, and the anchor is deployed by twisting the activation knob clockwise until 3 clicks are heard and a hard stop is reached. The surgeon ensures the anchor is fully seated directly beneath the cortical bone by gently pulling on the sutures. In total, 3 sutures are present: 1 blue, 1.4‐mm suture tape and a pair of black‐and‐white–striped passing sutures. The knee is set to 30° of flexion and neutral rotation while the graft is marked at the site of fixation (Figure 4). The repair suture tape is then threaded through a free needle, and the graft is stitched in a Krakow configuration. The repair suture tape is then passed into the looped end of the passing suture, which is pulled through the anchor, thus securing the graft (Figure 5). Again, ensuring the knee is set to 30° of flexion and neutral rotation, the graft is tensioned in a knotless fashion. The excess graft is then sutured back on itself using interrupted #2 braided suture (ULTRABRAID, Smith & Nephew) (Figure 6).

### Final Assessment and Closure

With fixation complete, the knee is taken through a full range of motion to assess stability and confirm the absence of overconstraint. The pivot‐shift test is performed to evaluate rotational stability, ensuring adequate graft function. If satisfactory, the incision is irrigated thoroughly, and the IT band is closed using 0‐vicryl, followed by layered closure of the subcutaneous tissue and skin. A compressive dressing is applied, and the patient is placed in a postoperative hinged knee brace set at 0 to 90° of flexion for initial rehabilitation.

## DISCUSSION

LET has garnered increasing attention as a supplementary procedure to ACL reconstruction, particularly in high‐risk patient populations, including athletes, those with high‐grade pivot shifts and individuals undergoing ACL revision surgery. Recent studies have provided robust evidence supporting the efficacy of LET in reducing re‐rupture rates and enhancing rotational stability.^1^, ^2^, ^6^, ^7^, ^19^ Notably, the STABILITY trial investigated the addition of LET in ACL reconstruction among young and active individuals. The trial revealed that supplementing ACLR with LET significantly decreased the risk of graft failure from 11% to 4%, while also enhancing knee stability, as indicated by the pivot shift test.^2^ Furthermore, Sonnery‐Cottet et al.^19^ examined LET when performed using modified Lemaire or Ellison techniques. Their study showed that these techniques provided superior control of rotational instability in high‐risk athletes, resulting in a lower re‐injury rate when LET was combined with ACLR compared with ACLR alone.^19^ A recent systematic review highlighted the role of LET in the context of revision ACL surgery. The authors found that including LET in revision procedures reduced graft failure rates by nearly 50%, which was particularly evident among younger patients with high‐demand lifestyles.^7^ This enhanced stability is due to the significantly diminished tibial internal rotation that the procedure provides, the kinematics of which were outlined by Inderhaug et al. in 2017.^20^

The advent of knotless all‐suture anchor fixation has revolutionized the orthopaedic landscape due to their minimal footprint and superior properties. LET femoral fixation was typically achieved via tunnel drilling and utilization of interference screws, which increased the possibility of tunnel convergence when performing concomitant ACLR.^21^ A recently published biomechanical analysis of all‐suture anchor fixation compared with staple and interference screw found comparable graft stiffness and adequate load‐to‐failure across all three implants; however, the interference screw did have a significantly higher ultimate failure load.^3^ The 1.8 mm knotless all‐suture anchors in particular have shown remarkable performance in biomechanical evaluations.^3^, ^14^, ^15^, ^16^, ^22^ Smith et al. compared all‐suture anchors to interference screws and interference suture anchors for medial patellofemoral ligament reconstruction in porcine knees and concluded that all‐suture anchor fixation had comparable cyclic elongation to that of interference fixation. The minimal footprint additionally eliminates the possibility of tunnel convergence.^16^, ^21^ Perhaps the greatest advantage of this technique over others utilizing knotless all‐suture anchor fixation in LET is the addition of 1.4 mm suture tape. Knotless fixation with suture tape has been shown to be biomechanically superior to constructs requiring tied knots as well as those with knotless fixation with #2 suture.^23^ While future clinical studies evaluating outcomes, failure rates, and functional recovery are needed to fully validate the proposed technique, the improved biomechanics of the 1.8 mm knotless all‐suture anchor with suture tape offers surgeons a reliable and replicable fixation strategy for LET.

While the LET is not necessary in all cases of ACL insufficiency, high‐risk patients including those with high‐grade pivot shift, hyperextension >10°, or generalized ligamentous laxity or those undergoing revision ACLR may be good candidates for the procedure. The modified Lemaire LET technique with 1.8 mm knotless all‐suture anchor fixation with suture tape represents an effective, minimally invasive approach to augment ACL reconstruction (Tables 1 and 2).

**TABLE 1:** Pearls and Pitfalls of a Lateral Extra‐articular Tenodesis with Iliotibial Band Using Knotless All‐Suture Anchor Femoral Fixation

Pearls	Pitfalls
• Preserve the Gerdy's tubercle attachment site by harvesting 1 cm strip of ITB distal to the lateral epicondyle extending 2 to 3 cm proximally	• Inadequate graft harvesting: a graft too short (<8 cm) may not provide sufficient length for fixation
• Achieve femoral isometric point by anchoring the tenodesis graft 8 to 10 mm proximally and posterior to LCL attachment	• Incomplete deployment of knotless anchor at femoral attachment site may result in graft slippage
• Support anterolateral ligament function via an extra‐articular tissue plane between LCL and lateral capsule to help pass the graft	• Inadvertently locking the sutures during Krakow stitching can prevent graft from sliding
• Perform pivot‐shift test intraoperatively to assess tension ensuring tibial internal rotation and knee flexion are properly balanced	• Excessive tensioning and non‐isometric anchor placement can lead to restricted knee motion and graft failure

ITB, ilitiobial band; LCL, lateral collateral ligament.

**TABLE 2:** Advantages and Disadvantages of a Lateral Extra‐articular Tenodesis with Iliotibial Band Using Knotless All‐Suture Anchor Femoral Fixation

Advantages	Disadvantages
• Increased strength of fixation with suture tape compared with techniques utilizing traditional suture	• Requiring good bone quality for proper fixation
• Minimally invasive with reduced soft tissue trauma	• Comparably less initial fixation strength to screw or button fixation
• Low‐profile design decreases hardware prominence and irritation	• Less suitable for large or high‐tension grafts
• Reduced risk of tunnel convergence	• Anchor failure may be harder to detect early

## DISCLOSURES

The author declares the following financial interests/personal relationships which may be considered as potential competing interests: M.B.B. reports a relationship with Vericel Corporation that includes consulting or advisory, reports a relationship with Smith & Nephew Inc., and reports a relationship with Stryker that includes consulting or advisory. The other authors (B.R.K. and S.S.) declare that they have no known competing financial interests or personal relationships that could have appeared to influence the work reported in this article.

## References

[1] Park YB , Lee HJ , Cho HC , Pujol N , Kim SH . Combined lateral extra‐articular tenodesis or combined anterolateral ligament reconstruction and anterior cruciate ligament reconstruction improves outcomes compared to isolated reconstruction for anterior cruciate ligament tear: A network meta‐analysis of randomized controlled trials. Arthroscopy. 2023;39:758‐776.e10. 36567183 10.1016/j.arthro.2022.11.032

[2] Getgood AMJ , Bryant DM , Litchfield R , et al. Lateral extra‐articular tenodesis reduces failure of hamstring tendon autograft anterior cruciate ligament reconstruction: 2‐year outcomes from the STABILITY study randomized clinical trial. Am J Sports Med. 2020;48:285‐297. 31940222 10.1177/0363546519896333

[3] Tollefson LV , Shoemaker EP , Slette EL , et al. Adequate failure loads for modified Lemaire lateral extra‐articular tenodesis are achieved with an interference screw, staple, and suture anchor: A biomechanical study of structural properties. Am J Sports Med. 2025;53:327‐332. 39760525 10.1177/03635465241305739

[4] Arora M , Shukla T , Garg S , Jani C . Functional outcomes and return to sport of combined ACL reconstruction and lateral extra‐articular tenodesis in high‐pivot Kabbadi players: A prospective cohort study of 93 elite players. Indian J Orthop. 2024;58:1626‐1634. 39539334 10.1007/s43465-024-01230-3 PMC11554599

[5] Bezawada PR , Vemula K , Yadlapalli SP . The functional outcome of lateral extra‐articular tenodesis combined with anterior cruciate ligament reconstruction in high‐risk patients: A prospective observational study. J Med Sci. 2024;10:188‐191.

[6] Boksh K , Mishra P , Akram N , Abdolrazaghi S , Singh H . Medial ulnar collateral ligament repair with augmentation: A systematic review and meta‐analysis of preclinical studies. Orthop J Sports Med. 2023;11:23259671231158373. 37152548 10.1177/23259671231158373 PMC10159257

[7] Grassi A , Olivieri Huerta RA , Lucidi GA , et al. A lateral extra‐articular procedure reduces the failure rate of revision anterior cruciate ligament reconstruction surgery without increasing complications: A systematic review and meta‐analysis. Am J Sports Med. 2024;52:1098‐1108. 38294248 10.1177/03635465231173698 PMC10943615

[8] Green DW , Hidalgo Perea S , Brusalis CM , Chipman DE , Asaro LA , Cordasco FA . A modified Lemaire lateral extra‐articular tenodesis in high‐risk adolescents undergoing anterior cruciate ligament reconstruction with quadriceps tendon autograft: 2‐year clinical outcomes. Am J Sports Med. 2023;51:1441‐1446. 36917840 10.1177/03635465231160681

[9] Slette EL , Mikula JD , Schon JM , et al. Biomechanical results of lateral extra‐articular tenodesis procedures of the knee: A systematic review. Arthroscopy. 2016;32:2592‐2611. 27324970 10.1016/j.arthro.2016.04.028

[10] Delaloye JR , Hartog C , Blatter S , et al. Anterolateral ligament reconstruction and modified Lemaire lateral extra‐articular tenodesis similarly improve knee stability after anterior cruciate ligament reconstruction: A biomechanical study. Arthroscopy. 2020;36:1942‐1950. 32251683 10.1016/j.arthro.2020.03.027

[11] Pearce SL , Bryniarski AR , Brown JR , et al. Biomechanical analysis of tibial motion and ACL graft forces after ACLR with and without LET at varying tibial slopes. Am J Sports Med. 2023;51:2583‐2588. 37462690 10.1177/03635465231184389

[12] Erdmann J , Pękala P , Zabrzyński J . A sonographic examination of the iliotibial band strip used in the mini‐open modified lemaire lateral extra‐articular tenodesis in patients with primary and revision ACL reconstruction—A pilot study. Appl Sci. 2025;15:4702.

[13] Behrendt P , Fahlbusch H , Akoto R , et al. Comparison of onlay anchor fixation versus transosseous fixation for lateral extra‐articular tenodesis during revision ACL reconstruction. Orthop J Sports Med. 2023;11:23259671231166380. 37213658 10.1177/23259671231166380 PMC10196542

[14] Darville GL , Young BL , Lamplot JD , Xerogeanes JW . Arthroscopic‐assisted lateral extra‐articular tenodesis with knotless anchor fixation. Arthrosc Tech. 2023;12:e2257‐e2264. 38196854 10.1016/j.eats.2023.07.054 PMC10772996

[15] Haus A , Chand A , Dawson K , Lang S , Gilmer BB , Wahl CJ . Modified Lemaire lateral extra‐articular tenodesis using an inlay technique and all‐suture knotless anchor fixation. Arthrosc Tech. 2023;12:e1607‐e1613. 37780650 10.1016/j.eats.2023.05.004 PMC10533872

[16] Temperato J , Ewing M , Nuelle CW . Lateral extra‐articular tenodesis with iliotibial band using knotless all‐suture anchor femoral fixation. Arthrosc Tech. 2023;12:e677‐e682. 37323783 10.1016/j.eats.2023.01.004 PMC10265525

[17] Maia Dias C , Gonçalves SB , Completo A , et al. Why are tapes better than wires in knotless rotator cuff repairs? An evaluation of force, pressure and contact area in a tendon bone unit mechanical model. J Exp Orthop. 2021;8:9. 10.1186/s40634-020-00321-y PMC7859138 33537914

[18] Suh DK , Kang MW , Kim TJ , Kim SY , Wang JH . Incidence of convergence between distally and anteriorly oriented ALL femoral tunnels and ACL femoral tunnels in combined ACL and ALL reconstruction: 3‐Dimensional computed tomography analysis of 227 patients. Am J Sports Med. 2024;52:902‐908. 38353108 10.1177/03635465241227223

[19] Sonnery‐Cottet B , Saithna A , Cavalier M , et al. Anterolateral ligament reconstruction is associated with significantly reduced ACL graft rupture rates at a minimum follow‐up of 2 years: A prospective comparative study of 502 patients from the SANTI study group. Am J Sports Med. 2017;45:1547‐1557. 28151693 10.1177/0363546516686057

[20] Inderhaug E , Stephen JM , El‐Daou H , Williams A , Amis AA . The effects of anterolateral tenodesis on tibiofemoral contact pressures and kinematics. Am J Sports Med. 2017;45:3081‐3088. 28763623 10.1177/0363546517717260

[21] Jaecker V , Ibe P , Endler CH , Pfeiffer TR , Herbort M , Shafizadeh S . High risk of tunnel convergence in combined anterior cruciate ligament reconstruction and lateral extra‐articular tenodesis. Am J Sports Med. 2019;47:2110‐2115. 31194569 10.1177/0363546519854220

[22] Smith BL , Bedi A , Hauck OL , Wijdicks CA , Riboh JC . All‐suture anchor onlay fixation for medial patellofemoral ligament reconstruction: A biomechanical comparison of fixation constructs. Orthop J Sports Med. 2024;12:23259671241294011. 39583149 10.1177/23259671241294011 PMC11585034

[23] Denard PJ , Adams CR , Fischer NC , Piepenbrink M , Wijdicks CA . Knotless fixation is stronger and less variable than knotted constructs in securing a suture loop. Orthop J Sports Med. 2018;6:2325967118774000. 29845084 10.1177/2325967118774000 PMC5964856

